# Influence of learning on range expansion and adaptation to novel habitats

**DOI:** 10.1111/j.1420-9101.2009.01836.x

**Published:** 2009-11

**Authors:** M SUTTER, T J KAWECKI

**Affiliations:** * Institute of Zoology, University of BaselBasel, Switzerland; † Department of Ecology and Evolution, University of LausanneLausanne, Switzerland

**Keywords:** biological invasions, heterogeneous environments, local adaptation, marginal habitats, niche evolution, plasticity, source-sink populations

## Abstract

Learning has been postulated to ‘drive’ evolution, but its influence on adaptive evolution in heterogeneous environments has not been formally examined. We used a spatially explicit individual-based model to study the effect of learning on the expansion and adaptation of a species to a novel habitat. Fitness was mediated by a behavioural trait (resource preference), which in turn was determined by both the genotype and learning. Our findings indicate that learning substantially increases the range of parameters under which the species expands and adapts to the novel habitat, particularly if the two habitats are separated by a sharp ecotone (rather than a gradient). However, for a broad range of parameters, learning reduces the degree of genetically-based local adaptation following the expansion and facilitates maintenance of genetic variation within local populations. Thus, in heterogeneous environments learning may facilitate evolutionary range expansions and maintenance of the potential of local populations to respond to subsequent environmental changes.

## Introduction

Learning allows an animal to modify its behaviour in an adaptive way in response to sensory feedback. A learning animal can compensate for the inadequacies of its genotype with respect to its current environment, and even develop an adaptive response to a novel situation, never encountered in the evolutionary past of the species. Learning will thus often modify the relationship between the genotype and fitness, and so affect the response to natural selection. More than a century ago, Baldwin (1896) and Osborn (1896) postulated that learning may facilitate response to directional selection. This phenomenon has indeed been observed in several mathematical and computer models (Hinton & Nowlan, 1987; Fontanari & Meir, 1990; Behera & Nanjundiah, 1995) and in an evolutionary experiment with *Drosophila* (Mery & Kawecki, 2004). However, other models predicted that learning should rather slow down the genetically-based responses to selection, in accordance with the intuitive notion that learning buffers the animal against the consequences of being genetically maladapted to its environment (Keesing & Stork, 1991; Papaj, 1994; Anderson, 1995; Mayley, 1997; Ancel, 2000; Dopazo *et al.*, 2001). A recent analytical treatment (Paenke *et al.*, 2007) suggests that, for learning to accelerate the response to directional selection, the gain in relative fitness brought by learning must be proportionally greater in genotypes that would already be fitter without learning. This requires rather special assumptions about the learning process and/or the shape of the relationship between phenotype and fitness (Paenke *et al.*, 2007). Another recent model suggests that learning may help a population to cross a ‘valley’ of low fitness in a complex adaptive landscape and to reach the domain of attraction of a higher ‘adaptive peak’ (Borenstein *et al.*, 2006). Learning-based flexible diet choice has also been predicted to drive the evolution of genetically-based resource specialization and to facilitate coexistence of species feeding on the same set of fully substitutable resources (Rueffler *et al.*, 2007). Finally, Beltman & Metz (2005) showed that learning-based imprinting on the natal habitat favours divergent habitat specialization, possibly facilitating sympatric speciation.

Except for Beltman & Metz (2005), the models cited above focus on evolution in homogeneous environments, and none incorporates an explicit spatial setting. In this paper, we address the effect of learning on expansion and adaptation of a species to a novel habitat, connected by dispersal (and so by gene flow) with the original (‘core’) habitat. The ability to adapt to novel, initially marginal habitats is a major factor affecting the dynamics of species distributions over evolutionary time. Adaptation to marginal habitats is thought to be often constrained by gene flow from core habitats, to which the species is already well adapted (Holt & Gaines, 1992; Kawecki, 1995, 2008). Because the species is initially poorly adapted to the novel habitat, the local population will often be a demographic sink, characterized by net immigration from source (core) habitats (Pulliam, 1988). On the one hand, this immigration may be necessary for the population in the novel habitat to persist. Even if this is not the case, immigration will augment the local population size and thus increase the number of individuals exposed to selection in the novel habitat. On the other hand, those immigrants continue bringing with them alleles at the frequencies typical of the core habitat, thus counteracting changes in the local gene pool brought about by selection in the novel habitat. While gene flow in general tends to hinder local adaptation, its effect is disproportionate in marginal habitats, in which the local populations are small and immigration exceeds emigration. Still another effect of the gene flow is to replenish genetic variation in marginal habitats. A number of theoretical studies (reviewed in Kawecki, 2008) have addressed the factors affecting adaption to such initially marginal habitats. They predict that the propensity to adapt is negatively correlated with the initial absolute fitness (i.e. lifetime reproductive success) in the marginal habitat, in particular if the initial performance is too low to sustain a local population without immigration (e.g. Holt & Gomulkiewicz, 1997; Kawecki, 2000). Adaptation to marginal habitats is also hindered by genetic architecture involving many loci with small effects (Holt & Gomulkiewicz, 1997; Kawecki, 2000, 2004), and if the fitness trade-off between the habitats is convex such that a small initial increase of fitness in the marginal habitat is associated with a large decrease in the core habitat (Holt & Gaines, 1992; Kawecki, 2000, 2003; Ronce & Kirkpatrick, 2001). The predicted relationship between the propensity to adapt and the dispersal rate are highly sensitive to assumptions (for a review and discussion, see Kawecki, 2008).

One mechanism by which learning may affect expansion into and adaptation to a novel, initially marginal, habitat is the one operating in the models reviewed in the first paragraph – learning may alter the relationship between the genotype and the relative fitness within the novel habitat. However, by affecting the mean absolute fitness (i.e. the net reproductive rate) in the novel habitat, learning will also affect the demography of the local population, with potentially important consequences for adaptive evolution. In particular, learning may allow a breeding population in the novel habitat to persist even if it is genetically so locally maladapted that without learning it could not persist even with immigration. More generally, by improving the survival and/or reproduction in the marginal habitat after its colonization, learning will tend to enhance the size of the local population, reduce the proportional contribution of immigrants to the local gene pool, and increase the contribution of the marginal population to the gene pool of the core population. All these factors have been predicted to facilitate adaptation to marginal habitats (Kawecki, 2008). Obviously, learning may also contribute to the fitness of individuals living in the core habitat. However, because the core population has (by definition) a long history of evolution in the core habitat, it is expected to be genetically well adapted, and thus the effects of learning on its mean fitness, and so on its demography, will usually be smaller. Learning may therefore facilitate expansion and adaptation to a novel, initially marginal habitat independently of its effects on the relationship between genotype and relative fitness.

Here, we use a spatially explicit individual-based model to study the effect of learning on adaptation to a novel habitat by a species initially adapted to another (core) habitat. The trait mediating fitness is resource preference, whereby the preference initially shown by each individual is genetically determined and can be subsequently modified within its lifetime by experience. The results indicate that learning may substantially increase the range of parameters under which the population successfully expands and adapts to the novel habitat, particularly if the border between the habitats is sharp. However, for a range of parameters, for which the population expands into the novel habitat even without learning, learning slows down genetically-based adaptation following the expansion and reduces the degree of local adaptation at equilibrium. It also facilitates maintenance of genetic variance.

## The model

We consider a one-dimensional, spatially explicit environment. To avoid edge effects, we assume that the environment space forms a ring: if an individual leaves the habitat space on the right, it re-enters on the left and vice versa. The environment contains two distinct habitats: the core habitat and the novel habitat. The habitats contain two different resources, which have the same abundance, but their quality *Q* varies from 0.5 to 1.5. The quality can be thought of as the amount of energy per unit resource consumed. In the core habitat, resource B has a quality of 1.5, whereas resource A has a quality of only 0.5, the opposite holds for the novel habitat. The two habitats have the size of 100 spatial units each, and are connected by two linear resource quality gradients (resource A and resource B) with the slope *m* and -*m*, respectively, the total size of the environment being 200 + 2/*m* units. Thus, at any point *x* in the habitat space, the qualities of the two resources satisfy: 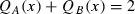
(1)

Both resources are assumed to be present in abundance, so that the choice is not restricted and it is always best to show complete preference for the resource which is locally of better quality. Note that 1/*m* is the length of the gradient (i.e. the distance between the two habitats).

The organisms living in this environment are diploid and carry eight unlinked loci, coding the innate (initial) preference *z*_0_ for resource A vs. resource B. Each locus has two alleles, allele ‘0’ increases the preference for resource B and allele ‘1’ for resource A, respectively. The innate preference of a given individual for resource A is given by: 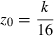
(2) where *k* is the number of ‘1’ alleles in the individual’s genome (i.e. the loci have equal and additive effect on preference). The innate preference determines the probability of choosing resource A at the first feeding round (see below).

Generations are discrete and the life cycle consists of four stages: (1) foraging, (2) viability selection and population regulation, (3) mating and reproduction, and (4) dispersal of the offspring.

During the foraging stage, each individual collects 10 food items (i.e. has 10 feeding rounds). The probability that resource A is chosen at the *i*th feeding round (*i* = 0, 1,…, 9) equals *z*_*i*_; resource B is chosen with probability 1 − *z*_*i*_. The initial (innate) preference *z*_0_ is determined genetically (see above). For the subsequent nine rounds, the preference may be modified by learning, based on experience of resource quality from the previous rounds. The average quality of the two resources is 1 at all points in space (see above). Thus, if the quality of the last resource item was greater than 1, the individual should increase its preference for this type of resource; if the quality was lower than 1 the individual should increase its preference for the other resource. Following the Rescorla–Wagner model of learning (Rescorla, 1988), we assume that the degree to which preference is modified following a single experience event increases with the disparity between the experience and the current preference (which in a sense reflects the individual’s expectation as to which resource should be better). Thus, if an individual with a strong preference for A finds resource A being of high quality (or resource B being of low quality), its preference towards A will increase only slightly, if only because the preference cannot be greater than 1, so there is not much scope left for the increase. In contrast, the same experience of finding a good A item (or a poor B item) will be more salient for an individual with current preference for B (a ‘surprise effect’, Rescorla, 1988), so the shift of this individual’s preference will be greater. The magnitude of the shift in preference will also depend on the individual’s learning ability, quantified as the learning rate *L*.

These assumptions are implemented in the following recurrence equations. If the item collected by an individual residing at spatial location *x* at round *i* was of resource A then the preference *z* for this resource changes according to 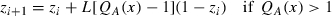
(3a)
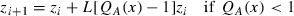
(3b)

If the item collected at the *i*th round was of resource B, the preference changes according to analogous equation obtained by replacing *z* with 1 − *z* and *Q*_*A*_ with *Q*_*B*_. The amount of resources *R* collected by an individual throughout the foraging phase equals the sum of qualities *Q* of the 10 food items, and thus ranges from 5 to 15.

Selection and population regulation act after foraging. The probability of survival until reproduction *S* is determined by the local population density *N*(*x*) and the amount of resources *R* collected by the individual during the foraging phase: 
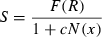
(4)

The local population density *N*(*x*) is the number of neighbours living within the distance of 0.5 units of point *x* (counted at the end of the foraging period); *c* is a scaling parameter that determines the carrying capacity. To keep the overall population size manageable, *c* was set to 0.15 for the simulations with abrupt transition between the habitats (*m* = ∞) and with a steep gradient (*m* = 0.1); for a shallow gradient (*m* = 0.01) *c* = 0.2 was assumed. The main effect of larger *c* is a lower population density per unit of space (results not shown). Because density-dependence is assumed to act independently of (i.e. multiplicatively with) the resource-dependent component, changes in *c* should have no systematic influence on the response to selection.

The resource-dependent component of survival *F*(*R*) is assumed to follow 
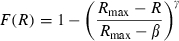
(5) if *R* > *β*; otherwise *F*(*R*) = 0. *β* is thus the minimum resource amount needed to survive and *R*_max_ = 10 max[*Q*_*A*_(*x*),*Q*_*B*_(*x*)] is the maximum amount of resources possible for the individual to collect. *γ* determines whether the relationship between *R* (for *R* > *β*) and survival is linear (*γ* = 1), convex (0 < *γ* < 1) or concave (*γ* > 1). That is, if *γ* ≪ 1, a large amount of resources above *β* has to be collected for a noticeable increase of survival.

The individuals are assumed to be outcrossing hermaphrodites. Each surviving individual acts once in the female (maternal) role and produces five offspring. The mating partner (i.e. the father) is chosen by selecting the spatially nearest of 100 randomly chosen individuals from the whole population; the same individual can act several times in the male role. Thus, mating occurs relatively locally, but not necessarily with the nearest neighbour. The genotypes of gametes are created through random recombination of the parental genotypes, assuming no linkage. The genome of an offspring mutates with the probability *μ* = 0.01 per genome. If a mutation occurs, a randomly chosen allele switches to the alternative state (i.e. from ‘0’ to ‘1’ or from ‘1’ to ‘0’).

The offspring disperse a random distance from the maternal parent. The dispersal distance follows a normal distribution with mean 0 and variance 1. Thus, less than 5% of individuals disperse farther than 2 spatial units, and less than 0.2% farther than 3 units. The adults die after reproduction.

The above assumptions were implemented in an individual-based stochastic simulation model, which thus implicitly incorporated demographic stochasticity and drift operating at several stages in the life cycle. Each simulation run started with 1000 individuals randomly distributed in the core habitat. This initial population was fixed for allele ‘0’ at all loci, i.e. showed complete innate preference for resource B (*z*_0_ = 0), and so was perfectly adapted to the core habitat, in which resource B was of higher quality. Discrete generations were implemented by carrying out each stage of each generation for all individuals before proceeding to the next stage. The total breeding population size in runs in which invasion of the novel habitat did not occur was about 1500, and reached about double that size if the invasion and adaptation to the novel habitat did occur.

We used the simulations to study the effect of learning and other parameters on the invasion of and adaptation to the novel habitat. We therefore varied the learning parameter *L* (from 0 to 0.6 in steps of 0.1), but also the parameters describing the selection (*β* from 1 to 12 in steps of 0.5 and *γ* from 0.1 to 2 in steps of 0.05) and the slope of the transition between the habitats (*m* = ∞, 0.1 or 0.01). The evolutionary expansion was considered successful if two criteria had been fulfilled before generation 5000: (1) the number of individuals living in the novel habitat had reached 99.99% of the local population size in the core habitat and (2) the mean innate (genetic) preference for resource A (mean *z*_0_) in the novel habitat reached 99.99% of the mean innate preference for resource B (1 − mean *z*_0_) in the core habitat. The first of these criteria reflects ecological success, the second genetic adaptation to the novel habitat. The mean innate preference (as well as its genetic variances which we also report in the Results) was calculated over individuals living within each habitat at least 10 units away from the habitat edges (the endpoints of the gradient). The census took place after selection (i.e. at the adult stage); the quantitative results concerning the mean and variance of *z*_0_ always refer to the adult population.

Simulations were run until the criteria for successful invasion were satisfied; if they had not been satisfied until generation 5000, the simulation was stopped. Between 1 and 27 simulation runs were carried out for each combination of parameters (a total of 69 462 simulation runs; average of 3.68 runs per parameter combination). The multiple simulation runs were concentrated in transition zones between regions of parameter space with different dynamics.

## Numerical analysis

### Trade-off in fitness between habitats

We first analyze numerically how learning affects the genetic trade-off in fitness between the core and novel habitat, mediated by the innate preference *z*_0_; this analysis sheds light on the simulation results. Because the effect of density-dependence on survival is independent of the genotype and phenotype (eqn 4), the expected value of *F*(*R*) is a measure of within-habitat relative fitness. Thus, the expected relative fitness conferred by a particular innate preference *z*_0_ at point *x* in space is 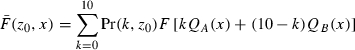
(6) where Pr(*k*, *z*_0_) is the probability that an individual with a particular *z*_0_ collects *k* items of resource A and 10 − *k* items or resource B; *F*(*R*) is defined in eqn 5. In the absence of learning (i.e. when *L* = 0), the probability of choosing resource A is *z*_*i*_ = *z*_0_ at each feeding round *i*, and so Pr(*k*, *z*_0_) follows a binomial distribution with parameter *z*_0_. If learning is permitted (*L* > 0), then the probability of choosing resource A changes from one feeding round to the next. The probability that the resource items collected by an individual with *z*_0_ follow a particular sequence (e.g. BBABAAABAA) can be calculated based on eqn 3 for all 10! sequences. Pr(*k*, *z*_0_) is then obtained as the sum of those probabilities for the 10!/[*k*!(10 − *k*)!] sequences with *k* items of resource A. Fitness conferred by a particular value of the innate preference *z*_0_ in the core habitat is obtained by substituting *Q*_*A*_(*x*) = 0.5 and *Q*_*B*_(*x*) = 1.5; in the novel habitat by substituting *Q*_*A*_(*x*) = 1.5 and *Q*_*B*_(*x*) = 0.5.

**Fig. 1:**
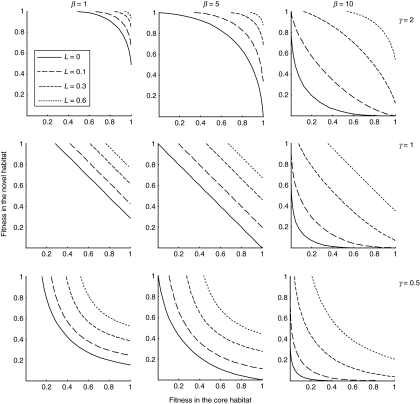
The effect of the selection and learning parameters on the genetic trade-off in fitness between the core and novel habitats, mediated by the genetically-based innate preference for resource A, *z*_0_. Each line shows the expected fitness in the two habitats (calculated based on eqn 6) as a parametric function of *z*_0_.

The resulting trade-off is plotted in Fig. 1 for several values of selection parameters *β* and *γ* (different panels) and a range of learning parameters *L* (different lines). Each line shows the two fitness values as parametric functions of *z*_0_, ranging from *z*_0_ = 0 (fitness in the core habitat equals 1) to *z*_0_ = 1 (fitness in the novel habitat equals 1). Recall that the minimum amount of resources collected by any individual is *R* = 5. Thus, if *β* ≤ 5, the individual fitness *F*(*R*) increases monotonically with *R* (eqn 5). In this case, the shape of the trade-off is determined by parameter *γ*: It is convex for *γ* < 1, linear for *γ* = 1 and concave for *γ* > 1; *β* determines the position of the trade-off line (Fig. 1). If *β* > 5, individuals which collect the amount of resources *R* < *β* have all zero survival. As a consequence, for *β* ≫ 5, the trade-off curve may be convex even if *γ* ≫ 1. Increasing the learning parameter has a similar effect as reducing *β*: for *β* ≤ 5, it shifts the trade-off curve towards higher fitness values without changing its shape; for *β* > 5, it additionally makes the curve less convex/more concave (Fig. 1, right panels). The endpoints of the trade-off line determine the fitness of a completely locally maladapted population, which has important consequences for the ability of such a population to persist and grow. The shape determines how much fitness in the core habitat is lost for an incremental improvement of fitness in the novel habitat. A convex trade-offs means that a small degree of adaptation to the novel habitat is associated with substantial loss of fitness in the core habitat, making adaptation to the novel habitat more difficult.

### Relationship between genotype and fitness

We also studied how learning affects the relationship between the innate preference *z*_0_ (i.e. the genotypic value) and log fitness in the novel habitat. According to Paenke *et al.* (2007), in a homogeneous environment, learning is expected to accelerate (slow down) response to directional selection if learning increases (reduces) the slope of this relationship. Numerical analysis indicated that under our assumptions about fitness and learning, and for the entire parameter range explored here, learning always flattens the slope of this relationship (for graphical illustration, see Figure S1). Thus, based on the argument by Paenke *et al.* (2007), learning would slow down the evolution in the adaptation to the novel habitat if the local population were cut off from immigration and still able to persist.

### Conditions for population persistence

**Fig. 2:**
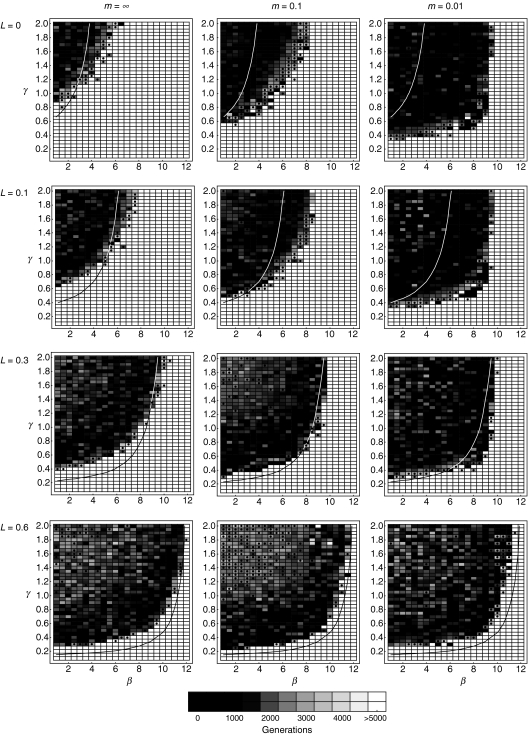
Time until adaptation to the novel habitat as a function of the selection parameters *β* and *γ* (axes of each panel), the steepness of the transition between the habitats (columns) and the learning rate parameter *L* (rows). The shading indicates the mean time in the simulations until both criteria for adaptation were satisfied; in the white area they were never satisfied before simulations were stopped at generation 5000. Black dots indicate parameter combinations for which the adaptation criteria were satisfied within 5000 generations in some but not all simulation runs; for those parameter sets the mean time to adaptation is calculated only over the runs in which the criteria were satisfied. The curves mark the combination of parameters at which the survival probability of individuals with *z*_0_ = 0 in the novel habitat equals 0.2; survivorship smaller than 0.2 implies that the population cannot persist without immigration (see text).

Finally, we used eqn 6 to determine the range of parameters under which even a population optimally adapted to the core habitat (*z*_0_ = 0) would have a positive expected growth rate at low density in the novel habitat. Several models suggested that the likelihood or degree of adaptation to a marginal habitat improves greatly when the initially maladapted population can persist without immigration (Kawecki, 2000; Ronce & Kirkpatrick, 2001; Kawecki & Holt, 2002; Holt *et al.*, 2003). It was thus interesting to see to what extent a positive expected growth of maximally maladapted population predicts the simulation results in this model. Because each surviving individual produced five offspring in its maternal role, the survival probability at low density must be greater than 0.2 to allow for population replacement. Thus, 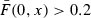 is a necessary condition for a population with *z*_0_ = 0 to persist in the novel habitat without immigration; otherwise, it would go deterministically extinct. This condition is satisfied for the values of *β* and *γ* to the left/above of the solid lines in the left panels in Fig. 2. Not surprisingly, learning increases the range of *β* and *γ* for which persistence of a genetically maladapted population in the novel habitat is possible without immigration (compare panels with different *L*). Note, however, that this condition being satisfied does not guarantee that the novel habitat will be colonized; particularly if the condition were just barely satisfied the local population would be particularly sensitive to demographic stochasticity.

## Simulation results

The density plots in Fig. 2 show the average time to adaptation to the novel habitat (according to the two criteria defined above) as a function of the selection parameters (*β* and *γ*), for a range of values of the learning rate (*L*; panel rows) and the slope of the gradient linking the habitats (*m*; panel columns). The most prominent feature of these plots is a rather sharp border between the region of the parameter space where adaptation never occurred within 5000 generations (white area), and the adjacent region where both criteria for adaptation were satisfied in most or all of the runs, usually within 2000 generations. Black dots indicate parameter combinations for which the criteria for adaptation were satisfied within 5000 generations in some but not all replicate simulation runs; in those cases, the average is calculated over the runs in which adaptation did occur.

### Dynamics of expansion

**Fig. 3:**
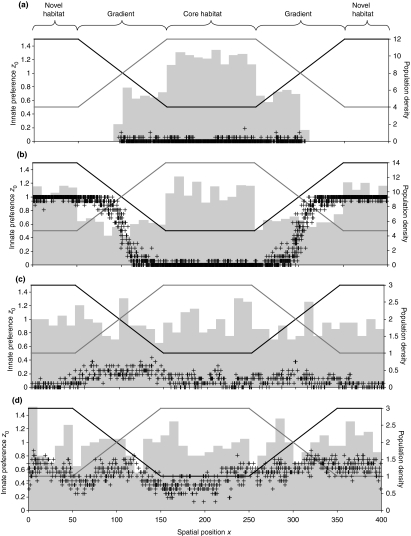
Snapshots of simulation runs: the distribution of individuals and their genetic (innate) preference for resource A. Each cross indicates the spatial position of an individual and its innate preference. The grey bars show population density as a number of individuals per unit of space (averaged over 10 units). The heavy black and grey lines indicate the quality of resource A and B, respectively. (a) Failure to expand into the novel habitat: *m* = 0.01, *L* = 0.3, *β* = 8.5, *γ* = 0.4, generation 5000. (b) Criteria for adaptation to the novel habitat satisfied: *m* = 0.01, *L* = 0.3, *β* = 8.5, *γ* = 0.4, generation 2310 (the time course of the simulation is shown in Animation S1). (c) Expansion into the novel habitat without local adaptation: *m* = 0.01, *L* = 0.6, *β* = 2, *γ* = 1.75, generation 100. (d) The same run as in panel (c) at generation 4800, for the time course see Animation S2.

Figure 3a illustrates a typical case where the population failed to expand into the novel habitat. The species was able to reach rapidly the carrying capacity in the core habitat and spread about half-way up the gradient, but was unable to cross permanently the point where resource A became better than resource B. Individuals dispersing beyond that point (or entering the novel habitat in cases with *m* = ∞) entered an area to which they were maladapted because of their strong innate preference for the locally inferior resource B. Thus, the local populations at the edge of the region with *Q*_*A*_ > *Q*_*B*_ were demographic sinks, largely sustained by immigration and with low population density (grey bars in Fig. 3a). Although in those marginal populations some evolution of the innate (genetically-based) preference towards less strong preference for resource B may be observed, in the white regions of Fig. 2, the average *z*_0_ remained well below 0.5. The failure to adapt meant that the marginal populations at the edges of species distribution could not increase in density and expand farther into the novel habitat. This conserved their status as demographic sinks.

Expansion and adaptation to the novel habitat despite the initial asymmetric gene flow involved a positive feedback between evolution and demography occurring at the expansion front. As the population adapted slightly, the local survival increased, and so the population could increase in density and expand in space, which in turn reduced the effect of gene flow on the local gene pool, making subsequent adaptation easier. For parameter values close to the edge of the dark regions in Fig. 2, the expansion process often seemed to ‘stall’ around the point where *Q*_*A*_ = *Q*_*B*_, sometimes for a long time, before this positive feedback started operating. Presumably, this latency reflected the need for the marginal population to reach, by demographic stochasticity and genetic drift, a favourable combination of local size and genetic composition that initiated the positive feedback. This was the case in the simulation run shown in Animation S1, in which the species expanded up to the points *Q*_*A*_ = *Q*_*B*_ on both sides of the core habitat within 100 generations, but only started expanding into the novel habitat after about 2100 generations, and only on the left hand side of the core habitat. It never crossed into the novel habitat on the right hand side of the core habitat; this region only became populated because the habitat space was assumed circular (Animation S1). In this run, the criteria for adaptation became satisfied in generation 2310; in another independent run with the same parameters, it only took 1250 generations; whereas in other eight runs, the species failed to invade the novel habitat within 5000 generations. Such a stochastic outcome – expansion and adaptation occurring in some runs but not in others – was typical for combinations of parameters adjacent to the region with no adaptation at all (indicated by black dots in Fig. 2).

### Factors favouring expansion

Inspection of Fig. 2 indicates that adaptation to the novel habitat was generally favoured by weak selection (small *β*, large *γ*), high learning ability (large *L*) and gradual transition between the habitats (small *m*). The effect of the selection parameters and the learning ability can be presumably mostly explained by their effect on the shape of the trade-off in fitness between the habitats (Fig. 1). Under small *β*, large *γ* and large *L*, the trade-off tends to be weaker (i.e. less convex or more concave) and the fitness of completely locally maladapted genotype tends to be higher (see ‘Numerical Analysis’ above). For the case of a sharp ecotone between the habitats (*m* = ∞, left column in Fig. 2), the region for which adaptations occurred most of the time roughly corresponds to the region where even a population with *z*_0_ = 0 would have a positive expected growth rate in the novel habitat (left of/above the curves in Fig. 2). However, the match is far from perfect. On the one hand, adaptation also occurred in a part of the region where population with *z*_0_ would not persist in the novel habitat without immigration (dark regions in Fig. 2 extend to the right of the curves; this is particularly prominent for *L* = 0). This is the region where the positive feedback between evolution and demography was essential for the expansion. On the contrary, the species failed to expand into the novel habitat for some parameter values for which even a population with *z*_0_ = 0 should theoretically have had a positive growth rate at low density (the white area extends to above the curve, particularly for high *L*). However, in our model, a positive expected growth rate was not a guarantee that the species would spread within the novel habitat. This was mostly because the poor growth potential of the marginal populations just beyond the habitat border was further reduced by density-dependence imposed by immigrants, even if they were completely maladapted. (Note that sink populations are per definition maintained above the density they would reach if dispersal were prevented; Kawecki, 2008).

Both high learning ability (large *L*) and a gradual transition between the habitats (small *m*) extended the range of selection parameters under which adaptation occurred, but their effects were not additive. The effect of learning was the strongest if the transition between the habitats was sharp (left column in Fig. 2). In turn the steepness of transition between the habitats made the greatest difference in the absence of learning (top row in Fig. 2). For a very gradual transition between the environment (*m* = 0.01, right column in Fig. 2), learning only had a small effect and vice versa.

While learning increased the range of selection parameters under which the species successfully expanded into the novel habitats, it slowed down adaptation for parameters under which this expansion would have occurred anyway. This can be seen in Fig. 2 as a lighter shade of squares in the upper-left corner of panels with *L* > 0. Additionally, for *L* = 0.6, a substantial proportion of simulation runs in that region of parameter space did not satisfy the adaptation criteria before generation 5000, as indicated by the prevalence of squares marked with dots in the upper-led quadrant of the bottom panels of Fig. 2. Note that for low *β*, high *γ* and high *L*, the trade-off in fitness between the habitats was virtually eliminated (see Fig. 1). Thanks to high learning ability even highly genetically maladapted individuals learned after a few feeding rounds to prefer resource A, and thus survived and reproduced well in the novel habitat. Therefore, the species colonized the entire novel habitat before any pronounced genetic adaptation became apparent (Fig. 3c, Animation S2). Because of the compensatory effect of learning, selection on the innate resource preference was weak (cf. Fig. S1), and so the genetic preference only evolved very slowly and was subject to large fluctuations due to drift (Fig. 3d, Animation S2). In contrast, in the absence of learning, the mean time until adaptation was quite similar across the range of parameters where adaptation did occur (Fig. 2, top row).

### Genetic differentiation and variance after expansion and adaptation

**Fig. 4:**
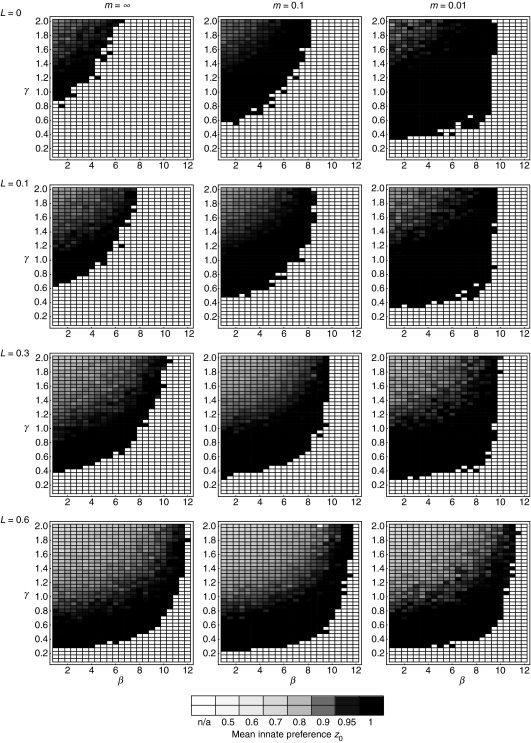
The degree of local adaptation after the criteria for adaptation to the novel habitat have been satisfied. The density plots show the mean innate (genetically-determined) preference for the locally better resource A in the novel habitat; according to the adaptation criteria the innate preference for resource B in the core habitat is essentially identical. Thus, the maximum possible degree of local adaptation corresponds to mean *z*_0_ = 1 whereas mean *z*_0_ = 0.5 would indicate a complete lack of genetic differentiation for the innate preference between the local populations in the two habitats. The mean preference was calculated over individuals present in the central part of the novel habitat, at least 10 distance units from the habitat edges. In the white area the adaptation criteria were never satisfied.

**Fig. 5:**
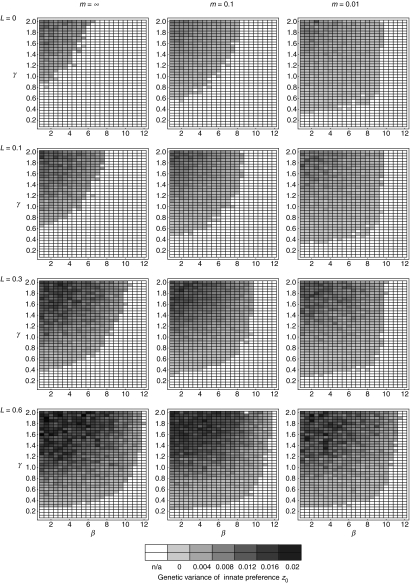
Genetic variance of the innate preference *z*_0_ within the novel habitat when the criteria for adaptation have been satisfied. The variance was calculated over individuals present in the central part of the novel habitat, at least 10 distance units from the habitat edges. In the white area the adaptation criteria were never satisfied.

The ability to learn considerably affected the degree of genetic differentiation of innate preference between the habitats and genetic variation within each habitat following a successful expansion and adaptation. Recall that the species was considered adapted to the novel habitat if both its population size and its mean innate preference for the locally better resource became as high in the novel habitat as in the core habitat. When a simulation reached these criteria, the system was presumably close to what in a corresponding deterministic model would be at equilibrium between local adaptation and gene flow. Examples of such an outcome are shown in Fig. 3b, d. At this quasi-equilibrium, the mean innate preference for resource A in the novel habitat indicates the degree of genetic differentiation between the habitats. (Recall that according to the adaptation criterion, the mean *z*_0_ in the novel habitat equals 1 − mean *z*_0_ in the core habitat; mean *z*_0_ equal 0.5 would indicate no differentiation.) This differentiation reflects the balance between local selection and gene flow, and so can be seen as a measure of local adaptation. Note that the degree of genetic differentiation between the local populations is much lower, and the genetic variation maintained within local populations larger, in Fig. 3d than in Fig. 3b. The simulation results (Fig. 4) indicate that the genetic differentiation is the strongest for parameters just at the edge of the region where adaptation occurs at all. In this region, the mean *z*_0_ in the novel habitat is often greater than 0.95, which implies mean *z*_0_ < 0.05 in the core habitat. In the absence of learning (*L* = 0), the degree of differentiation declines somewhat as *β* decreases and *γ* increases, with the mean *z*_0_ about 0.85–0.9 in the upper-left corner of the top row of panels in Fig. 4. This decline is much stronger when a high learning rate is assumed; with *L* = 0.6 the mean *z*_0_ in the novel habitat for low *β* and high *γ* can be as low as 0.6. This reduction in the degree of genetic differentiation between habitats brought by learning is accompanied by an increase in genetic variance within each habitat (Fig. 5). Thus, for a significant portion of the parameter space, learning reduces the degree of local adaptation and increases genetic variation within local populations. The slope of the transition between the habitats (*m*; columns in Figs 4 and 5) has little effect on the degree of genetic differentiation and the genetic variance within habitats.

## Discussion

In our simulations, the ability to learn significantly extended the range of selection parameters under which the species was able to expand and adapt to a novel habitat if the two habitats were separated by a sharp transition (Fig. 2). This can be attributed to two ways in which learning affected the trade-off in fitness between the habitats (Fig. 1). First, thanks to learning individuals adapted to the core habitat, i.e. having a strong genetic preference for resource B, could achieve a higher absolute fitness (survival probability) than they would have achieved if their preference had not been modified by experience. This extended the range of parameters where even a locally maladapted population could establish and persist in the novel habitat. Second, when the minimum amount of resources needed for a nonzero chance of survival (i.e. parameter *β*) was large, learning made the trade-off in fitness between the habitats less convex/more concave. This amounted to reducing the cost in terms of fitness in the core habitat of improving fitness in the novel habitat. Both effects enhanced the effectiveness of local selection in the novel habitat relative to gene flow from the core habitat.

The facilitating effect of learning on the expansion into a novel habitat can thus be attributed to its buffering effect on the absolute fitness differences between habitats. However, the buffering effect of learning also reduced the relative differences in fitness within the habitats. This made selection following the expansion into the novel habitat less efficient, with three consequences. First, for a broad range of parameters, the time until a comparable degree of adaptation was reached in both habitats increased with higher learning ability. Second, the final degree of local adaptation as measured by differentiation in mean innate preference between the habitats was substantially reduced. Third, learning facilitated the maintenance of genetic variation.

How robust are these conclusions likely to be? Obviously, we had to assume a particular model of genetic architecture, the learning process, foraging the demography. In particular, the fixed total number of resource items that an individual can collect implies that search time is negligible relative to the handling time. This could apply, e.g. to a bee exercising a choice between flower species that are present in abundance but present difficulties in extracting nectar. Alternatively, this assumption may represent a situation where search time is not negligible but simultaneous search for both resource times is precluded because the two resources occur in different microhabitats or require different search techniques. For example, a bird may have to choose between foraging in the canopy and in the understory. When such exclusive choices must be made, learning is particularly useful. Another situation that would favour learning in the context of diet choice is where tackling a particular resource type (prey) might actually be harmful or dangerous. For example, a predator of butterflies in South American rainforests must develop, through learning or evolution, avoidance of colour patterns characteristic of the local toxic *Helicomius* butterflies; these patterns vary strongly among regions (Joron & Mallet, 1998). While it is not directly implemented in our model, one can conjecture that under this scenario learning would have similar effect on range expansion and local adaptation. However, in many biologically relevant foraging scenarios assuming short handling times it pays to collect all encountered resource items, or at least the fitness cost of consuming a suboptimal item is small (Krebs & Davies, 1993). In such cases, learning ability would presumably have a lesser influence on evolutionary range expansions.

Furthermore, we assumed that the adaptation to the novel habitat was mediated by the very trait that was subject to learning. However, local adaptation will often be mediated by physiological, morphological or life history traits, which are not amenable to learning. Would our conclusions extrapolate to such cases? As argued above, the effect of learning in the initial phase of expansion into novel habitats seems to be largely mediated by a reduction in the demographic asymmetry between the habitats. This only requires that learning alleviates the poor survival and fertility suffered in the novel habitat by locally maladapted immigrants. If the novel habitat requires, say, a greater tolerance to cold, learning may still help warm-adapted immigrants to survive if it helps them find food – well-fed animals will often be more resistant to cold stress than malnourished ones. Thus, learning may facilitate the expansion even if the genetically-based adaptation is mediated by traits not amenable to learning. How learning under this scenario would affect the degree of local adaptation and the maintenance of genetic variance following expansion would depend on its effect on the relationship between the genotype and fitness within each habitat. Still, even if the phenotypic effect of learning on a particular trait (such as foraging efficiency) would be independent of the trait mediating local adaptation (such as cold tolerance), the fitness effects would not have to be independent. One can imagine circumstances under which individuals whose thermal tolerance is farther away from the local optimum would profit more in terms of fitness from learning-mediated improvement in foraging success. If so, being able to learn while foraging would diminish the effectiveness of local selection relative to gene flow. As in our simulations, this should lead to a lesser degree of local adaptation and more genetic variation being maintained within local populations.

A model by Beltman & Metz (2005) also addresses the effects of learning on habitat adaptation in a two-habitat system, although with no explicit spatial setting. Our results are difficult to compare with theirs because of several important differences in assumptions. First, they assume that learning affects habitat choice rather than the choice of resources within a habitat. Second, learning is not based on the perceived habitat quality, but involves imprinting on the natal habitat – it makes an individual more likely to choose the same type of habitat as the one in which it was born, even if its fitness would be higher in the other habitat. Third, rather than being a parameter, in their model, learning ability is an evolving trait, and the degree to which it evolved is affected by a parameter determining a fitness cost of learning. This cost of learning in their model has no systematic effect on the parameter range under which the population remained confined to a single habitat. This seems to contradict our results, but may be a consequence of the imprinting-type learning assumed by Beltman & Metz (2005). If the species evolves to occupy both habitats, they find that learning facilitates genetic differentiation of the local populations in the two habitats. This qualitative result again seems opposite to what our model predicted, but this again is likely a consequence of the type of learning assumed – in their model high learning automatically means high habitat fidelity and so genetic isolation (Beltman & Metz, 2005). While their model and our model are not directly comparable, both indicate that learning may have important consequences for adaptive evolution in heterogeneous environments and underline the need for more research in this direction.

Our own species is perhaps the best illustration of the consequences of behavioural flexibility for both ecological expansion and evolutionary adaptation to diverse habitats. On the one hand, thanks to our ability to learn, our species was able to establish persistent populations in all but the most extreme land environments. On the other hand, the degree of genetically-based local adaptation is rather limited, putative examples such as skin colour (Jablonski, 2004) or resistance to locally endemic diseases (Balter, 2005) notwithstanding. Rather, we compensate for the inadequacy of our physiology and anatomy with respect to the environment through products of civilization, relying on innovation and (social) learning (Laland & Brown, 2006). While humans are a special case and the above arguments are anecdotic, some comparative studies of other taxa hint on the role of learning in expansion into novel habitats. In particular, bird species with larger brains relative to their body mass and higher potential for innovative behaviour tend to be more successful at establishing themselves in novel environments (Sol *et al.*, 2002, 2005). A similar correlation between the relative brain size and invasiveness has been reported in mammals (Sol *et al.*, 2008). Nicolakakis *et al.* (2003) found a positive relationship across avian taxa between behavioural flexibility, measured as innovation frequency, and species richness. Given that most speciation events in birds are presumably allopatric, greater species richness is likely correlated with the ability to colonize geographically distant areas. Hence, the relationship between behavioural flexibility and species richness could be plausibly mediated at least in part by the potential to establish in novel habitats.

Although some models indicated that learning can accelerate the response to directional selection by magnifying the differences in relative fitness (Hinton & Nowlan, 1987; Fontanari & Meir, 1990; Behera & Nanjundiah, 1995), in homogeneous environments, the conditions for this are rather stringent (Ancel, 2000; Paenke *et al.*, 2007). Under most biologically relevant scenarios, learning will have a buffering effect, reducing the effective strength of selection. This is also the case for the form of learning assumed here. Yet, because of interplay between evolution and demography, in a spatially heterogeneous setting this buffering effect facilitated evolutionary expansions into a novel habitat. Thus, learning may after all be an important factor driving genetically-based evolutionary change in heterogeneous environments.

## Supporting information

**Figure S1** The effect of learning on the relationship between the innate preference for resource A and the log fitness in the novel habitat. (Including full legends for animations.)

**Animation S1** Simulation run from Fig. 3b. After being confined to the core habitat for a long time the population expands and simultaneously adapts to the novel habitat.

**Animation S2** Simulation run from Fig. 3c, d. A weak trade-off in fitness between the habitats allows the population to expand quickly into the new habitat; local adaptation evolves only slowly following the expansion.

As a service to our authors and readers, this journal provides supporting information supplied by the authors. Such materials are peer-reviewed and may be reorganized for online delivery, but are not copy-edited or typeset. Technical support issues arising from supporting information (other than missing files) should be addressed to the authors.
