# Supplementary material for: Influence of learning on range expansion and adaptation to novel habitats
Source: J Evol Biol. 2009 Nov;22(11):2201–14. doi: 10.1111/j.1420-9101.2009.01836.x (PMC2779469; doi:10.1111/j.1420-9101.2009.01836.x)
Supplement: Supplementary file 1 [file jeb0022-2201-SD1.pdf]

Influence of learning on range expansion and adaptation to novel habitats

Michael Sutter[1,3] and Tadeusz J. Kawecki[2,4]

Supporting Information

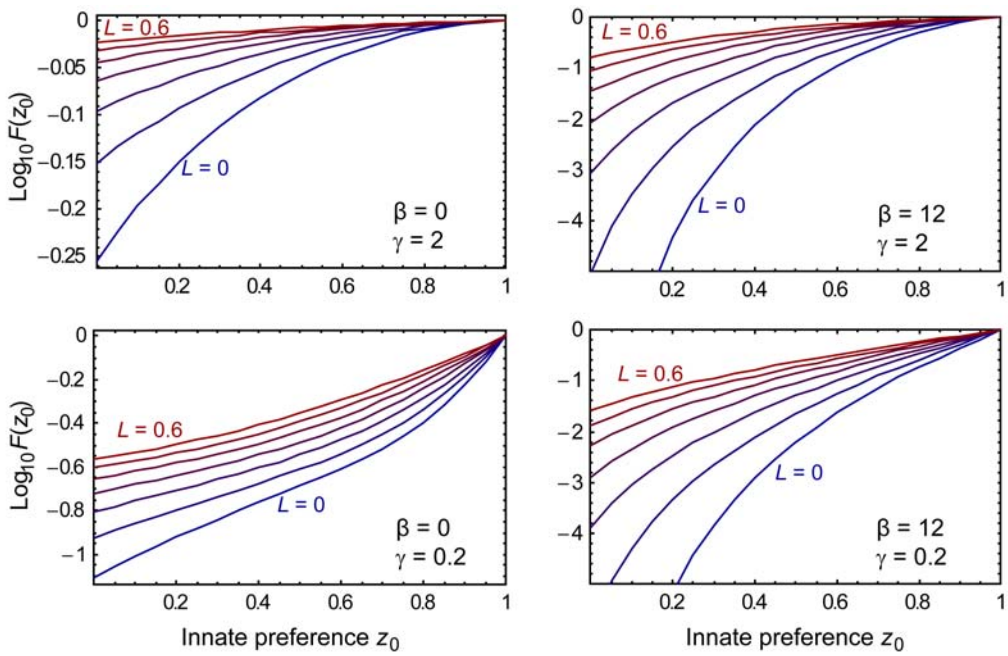

Figure S1. The effect of learning on the relationship between the innate preference for resource A and the log fitness in the novel habitat. The learning parameter *L* ranges from zero (i.e., no learning, blue lines) to *L* = 0.6 (red lines) in increments of 0.1. The combinations of selection parameters β and γ used in the four panels correspond to the four corners of the parameters space in each panel of figures 2, 4 and 5.

Description of animations

The dynamics of adaptation to a novel habitat: examples. Each individual is represented as a red asterisk, which indicates its genetic (innate) preference for resource A (z_0), and as a black dot, indicating its preference for resource A after 10 runs of foraging (z_{10}). The blue and green lines indicate the local quality of resource A and B, respectively.

Animation 1: simulation run from figure 3B. After being confined to the core habitat for a long time the population expands and simultaneously adapts to the novel habitat; *m* = 0.01, *L* = 0.3, β = 8.5, γ = 0.5; every 10th generation shown.

Animation 2: simulation run from figure 3C,D. A weak trade-off in fitness between the habitats allows the population to expand quickly into the new habitat; local adaptation evolves only slowly following the expansion; *m* = 0.01, *L* = 0.6, β = 2, γ = 1.75; every 100th generation shown
